# Clinical sentiment analysis of ICU notes: a dataset and a comparison of lexicon and clinical language model methods

**DOI:** 10.1186/s12911-026-03509-x

**Published:** 2026-05-18

**Authors:** Shahad Nagoor, Lucy Hederman, Kevin Koidl, Foad Bukhari, Sandra Chiaka Amasike, Antonie Bianchi, Chris Yen-Chen Lo, Hiral Patel, Ignacio Martin-Loeches

**Affiliations:** 1 School of Computer Science and Statistics, Trinity College Dublin, Dublin, Ireland; 2 Department of Computer Science, Faculty of Computing and Information Technology in Rabigh, King AbdulAziz University, Rabigh 21911, Saudi Arabia; 3 Department of Medicine, College of Medicine, Taibah University, Madinah, Saudi Arabia; 4 Intensive Care Medicine, St. James’s Hospital, Dublin, Ireland; 5 Department of Anesthesiology and Intensive Care Unit North Hospital, Aix Marseille University, Assistance Publique Hôpitaux Universitaires de Marseille, Marseille, France; 6 Intensive Care Units, Beaumont Hospital, Dublin, Ireland; 7 Department of Intensive Care Medicine, Multidisciplinary Intensive Care Research Organization (MICRO), St James’ Hospital, Dublin, Ireland; 8 School of Medicine, Trinity College Dublin, Dublin, Ireland; 9 Trinity Centre for Biomedical Engineering (TCBE), Dublin, Ireland

**Keywords:** Critical care, Clinical notes, Sentiment analysis, Clinical sentiment, Natural language processing, Generative artificial intelligence, Large language models

## Abstract

**Background:**

General-domain sentiment models have been found ineffective in distinguishing positivity and negativity in Intensive Care Unit (ICU) clinical notes and domain-specific sentiment models are recommended. Although there are multiple common approaches to sentiment analysis, there has been little work comparing and evaluating the effectiveness of specialized models, largely due to the difficulty of recruiting clinical annotators and of accessing clinical sentiment data. This study has three contributions: (1) **MIMIC-III-Ext-Notes-Sentiment:** the first public ICU-specific ground- truth dataset labeled by clinicians for investigating clinical sentiment polarity in ICU clinical notes. (2) **SentimentICUModel:** an effective model for classifying clinical sentiment in ICU narratives on the ground truth. (3) A guiding comparison of the effectiveness of a range of approaches to clinical sentiment classification on the dataset.

**Methods:**

We recruited five clinicians to annotate notes for the ground truth. Annotators indicated which pieces of note text influenced their labeling. Six clinical-specific models were compared on the ground truth.

**Results:**

The task of annotation was challenging due to clinicians’ workload and spanned 15 months. The ground truth data was formed based on inter-annotator agreement analysis. Clinicians’ extracts similarity aligned with their agreement level. Clinical language models provide comparable accuracy (up to 82%), with top score achieved by ClinicalT5 which is being released as SentimentICUModel. They outperform keyword-based lexicon (p$$ < 0.05$$, [95% CI, −0.47, −0.28]).

**Conclusion:**

Clinical language models have demonstrated effectiveness in identifying clinical sentiment within clinical notes, enabling early detection of sudden changes and exploring different patterns in patients’ ICU stays.

**Supplementary information:**

The online version contains supplementary material available at 10.1186/s12911-026-03509-x.

## Introduction

Clinicians in ICU spend valuable time documenting their clinical impressions and prognosis about patients in clinical notes. Impressions are essential in providing an overall understanding of patients’ status and progress. Capturing positivity or negativity about patients’ clinical status is an important factor in comprehending their progress in ICU stays, but can be exhausting and time-consuming especially for complicated cases. Therefore, an automatic negative/positive polarity identification for ICU notes will provide significant assistance in exploring their progress including onset of deterioration or sudden shifts in patient status and consequently support ICU clinicians’ decisions. Identifying such polarity has also been described as clinical sentiment^1^.

Clinical sentiment has been under-researched due to difficulty of accessing appropriate data and limited time that specialised clinicians can provide for preparing such data. Preparing manually annotated data by experts in the field is essential for AI supervised learning and automated analysis comparing machine vs. human performance. Specifically, a set of notes whose “sentiment” has been assigned manually by expert annotators is required. No such labeled datasets of ICU notes exist. Because clinical language is so specialized, the existing general domain models have not succeeded in recognizing clinical polarity [1, 2]. As a consequence, very little work and attention has been paid to sentiment in this specific domain, and the development of clinical-specific sentiment approaches, including lexicons and AI models, has not been progressed. Therefore, their capabilities and performance have not been explored. To address this gap, and based on the anticipated benefits, the aim of this research is to investigate **which among the specialized methods of sentiment analysis approaches can offer the best capture of clinical polarity in ICU notes?** On that basis, this research has three main contributions:

1. **MIMIC-III-Ext-Notes-Sentiment**^2^
2. **SentimentICUModel**^3^ the most effective model for classifying clinical sentiment in ICU narratives on the ground truth.
3. An exploratory comparison of the effectiveness of different approaches to clinical sentiment analysis including an existing recent ICU-specific lexicon, and clinical language models in clinical sentiment classification for ICU clinical notes.

## Related work

Our previous study [3] presents literature on clinical-specific lexicons and language models for sentiment analysis. Overall, despite the poor effectiveness of general domain models, most related work used them to capture clinical sentiment [1, 4], and predict important outcomes. e.g., risks of re-admission or mortality [5–7]. A recent study [8], concurrent to our work, targets this gap and developed an ICU specific sentiment lexicon, and investigated its predictive accuracy against an ML model. The study insists on the need for ICU specific sentiment tools, but their analysis is based on note fragments and only compares with a single BERT model (DeBERTa-v3). Our previous study [3] evaluated three lexicons, three BERT models and three T5 models, both general and clinical-specific, and used a larger scope of notes texts. This study extends that work as explained in the next section.

## Method

We studied six selected models from four families: a keyword-based ICU specific lexicon, a specialised BERT model (ClinicalBERT), two generative prompt models (ClinicalT5, SciFive which are specialised T5-based models), and two large clinical LLMs (medgemma-4b-it [9], and PMC_LLaMA_13B [10]).

We created three datasets, two from MIMIC-III data: a training dataset to train language models, and an evaluation dataset to evaluate the lexicon and the language models. Training data is automatically labeled using a labeling strategy suggested in [3], see section “Training Data”. The evaluation dataset is labeled by clinicians. In addition, we used an external COVID dataset [11] for evaluation.

**Fig. 1:**
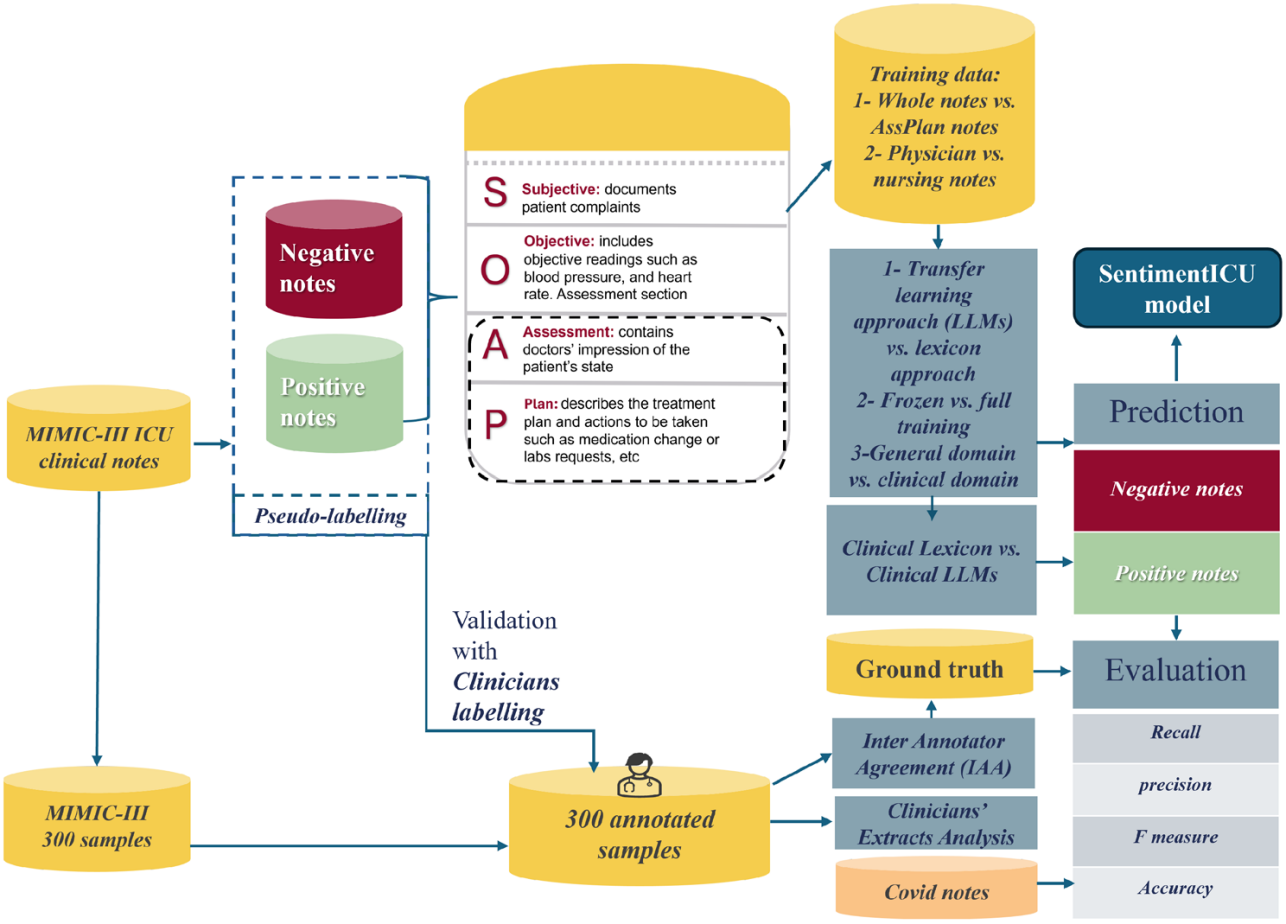
Research method

In summary, the steps involved in this research study are:

- Create automatically pseudo-labeled training data.
- Recruit annotators for manual annotation of evaluation data and collect their annotations.
- Study inter-annotator agreement and construct ground truth.
- Evaluate pseudo-labeled dataset based on ground truth.
- Train clinical language models using training data (pseudo-labeled) and evaluate them on ground truth data.
- Evaluate models on external data (COVID data)
- Explore the use of large LLMs on clinical sentiment with three-classes including no-sentiment and with zero and few shot settings.
- Analyse text extracts influencing annotators’ decisions using text similarity measures. Research method is summarized visually in Fig. 1.

### Datasets

The data used in this study is a subset of MIMIC-III, plus a COVID dataset [11]. MIMIC-III notes are written in English and contain de-identified information about patients in the intensive care units at Beth Israel Deaconess Medical Center in Boston, Massachusetts, USA in the period 2001–2012 [12–14]. We target physician and nursing progress notes, which belong to patients who stayed in ICU, due to their value and influence on prediction in critical care [15–17]. Additionally, based on [8] findings, these notes hold great proportions of sentiment words among other types (physician notes hold 97.8% while nursing hold 81.1% of sentiment keywords). A synthesized example of a note is shown in Supplementary A.

The COVID dataset [11] contains clinical notes associated with *chest X-ray* and *CT images* of patients infected with or suspected of COVID-19 or other pneumonia causes.

#### Training data

Training data is a collection of MIMIC-III notes, selected based on timing attributes of patients’ ICU stays and labeled using a specific automatic pseudo-labeling scheme. The labelling, introduced in [3], aligns with findings by [1]. Notes are labelled based on the assumption that patients are likely to be critically ill when admitted to ICU and near to mortality time (negative notes) and are likely to be improving when discharged to home (positive notes). In a previous study [3], informed by [18–21], we found that clinical sentiment of the part of a note from SOAP [22] Assessment point onward and physicians’ notes, achieves slightly better accuracy than the whole note, and nursing notes. In this study, we train models on each category separately to compare their effect. The training datasets is class-balanced and contain 10,766 notes of each category.

#### Manual annotation

Five^4^ annotators were recruited for the task of manual annotation of 300 notes. Recruiting clinicians was very challenging due to their workload (See section “Challenges and future work”). The annotators were an ICU consultant, a respiratory consultant, and three ICU registrars. Two meetings were held with each annotator prior to the task, the first to explain the MIMIC-III data access requirements, the second to explain the manual annotation task (see Supplementary B). For each note annotators were asked to choose a sentiment label by answering the following question: **Do you think that the person who wrote this note believes the patient’s health:**

- Is already improving or will get better?
- Is deteriorating?
- Is neither improving nor deteriorating?
- OR that there is no indication of the note writer’s opinion.

We study Inter-Annotator Agreement (IAA) using Fleiss kappa [23]. Results are presented in section “Results”.

#### Sampling

The MIMIC-based annotation dataset consists of 52% (156/300) physician notes and 48% (144/300) nursing notes. We ensured there were at least 20 samples from each of the three pseudo-labeling groups (i.e., notes after admission, prior to discharge home, and prior to death) so that we could compare the labeling validity with expert labeling (see section “Evaluation of pseudo-labeling”). We also included samples that do not belong to the three groups to study annotators’ agreements on such notes. Statistics relating to the manual annotation dataset are provided in Table 1.

**Table 1:** Statistics for the manually annotated dataset

Group	Counts
Patients	248
ICU stays	251
Patients with LOS $\le$6	107
Physicians’ notes	156
Nursing notes	144

From the COVID dataset, we extracted and annotated 23 notes of ICU patients with support of a respiratory consultant.

### Models

For full-fine-tuning, as described earlier in this section, we select three different clinical language models and an existing keyword-based lexicon (non-learning algorithm) to investigate their capabilities in capturing clinical sentiment polarity for ICU clinical notes. For zero and few shots, we use medgemma-4b-it, and PMC_LLaMA_13B as presented in section “Zero-shot and Few-shot learning by Clinical LLMs”.

The keyword lexicon is the keyword list from [8] which we will refer to as Kennedy’s lexicon. This list of 103 negative and 72 positive keywords and phrases was extracted and reviewed iteratively by a group of clinicians. Sentiment in their analysis was measured by a score calculated based on Eq. (1).

1 $$ \text {Sentiment score }=\frac{\text {NK }}{\text {NK }+ \text {PK }}$$

where NK is the number of negative keywords, and PK the number of positive keywords.

The three clinical transformers we use are ClinicalBERT [24] from BERT models family, SciFive [25], and ClinicalT5 [14, 26], both from the T5 family. ClinicalBERT and SciFive are open-source and publicly available in the Hugging Face library [27]; ClinicalT5 is available under protected access through PhysioNet website^5^. We described each model’s preprocessing requirements in [3]. ClinicalBERT and ClinicalT5 were trained on MIMIC-III text data^6^. SciFive was trained on abstracts and articles from PubMed and PubMedCentral. These three models have demonstrated the top accuracy scores in our previous work [3] against general domain lexicons and models. The aim in the previous study was to investigate the effect of domains on sentiment recognition and to have an initial assessment of the labeling strategy, hence all datasets were automatically (pseudo-) labeled (see section “Training Data”). In this work, we build on the findings and evaluate those models on a human-labeled dataset, providing (1) evaluation of automatic labeling vs. human labeling; and (2) evaluation of machine learning models performance vs. human performance.

In experimentation with clinical language models, we explored the following hyper-parameters: learning rate in the ranges: {1e-2 to 1e-5}, {3e-4, 3e-5}, and {5e-4, 5e-5}, batch size in the values: {4, 8, 16, 32, 64, 128}, and epochs in the range {1–10}. We applied early stopping and selected the best hyper-parameters, shown in Table 2.

**Table 2:** Experiment details for learning models. Bt = Batch, LR = Learning rate, Ep = Epochs, Opt = Optimizer, Tk = Token size

Model	Bt	LR	Ep	Opt	Tk
ClinicalT5	64	1e-3	8	AdamW	512
SciFive	64	1e-3	8	AdamW	512
ClinicalBERT	64	1e-4	4	AdamW	512

## Results

This section discusses the inter-annotator agreement in the manual annotation, construction of ground truth, validation of pseudo-labeling, evaluation of sentiment models on the annotated dataset, and annotators’ extracts of texts.

### Inter-annotator agreement

The overall agreement for all annotators over all 300 notes was slight using Fleiss’s Kappa (0.1863) based on the interpretation by this method [28]. Counts matrix for classes distribution by annotators is shown in Supplementary D.

The slight inter-annotator agreement is common even among very professional clinicians based on many previous studies [29], and could be explained by one or more of the following:

- Missing indicative details or implicit clinical sentiment [29–32].
- Differing focus [18].
- Inadequate attention [29, 32].
- Unfamiliar note style [21, 31].
- Different backgrounds [29, 33, 34].

**Fig. 2:**
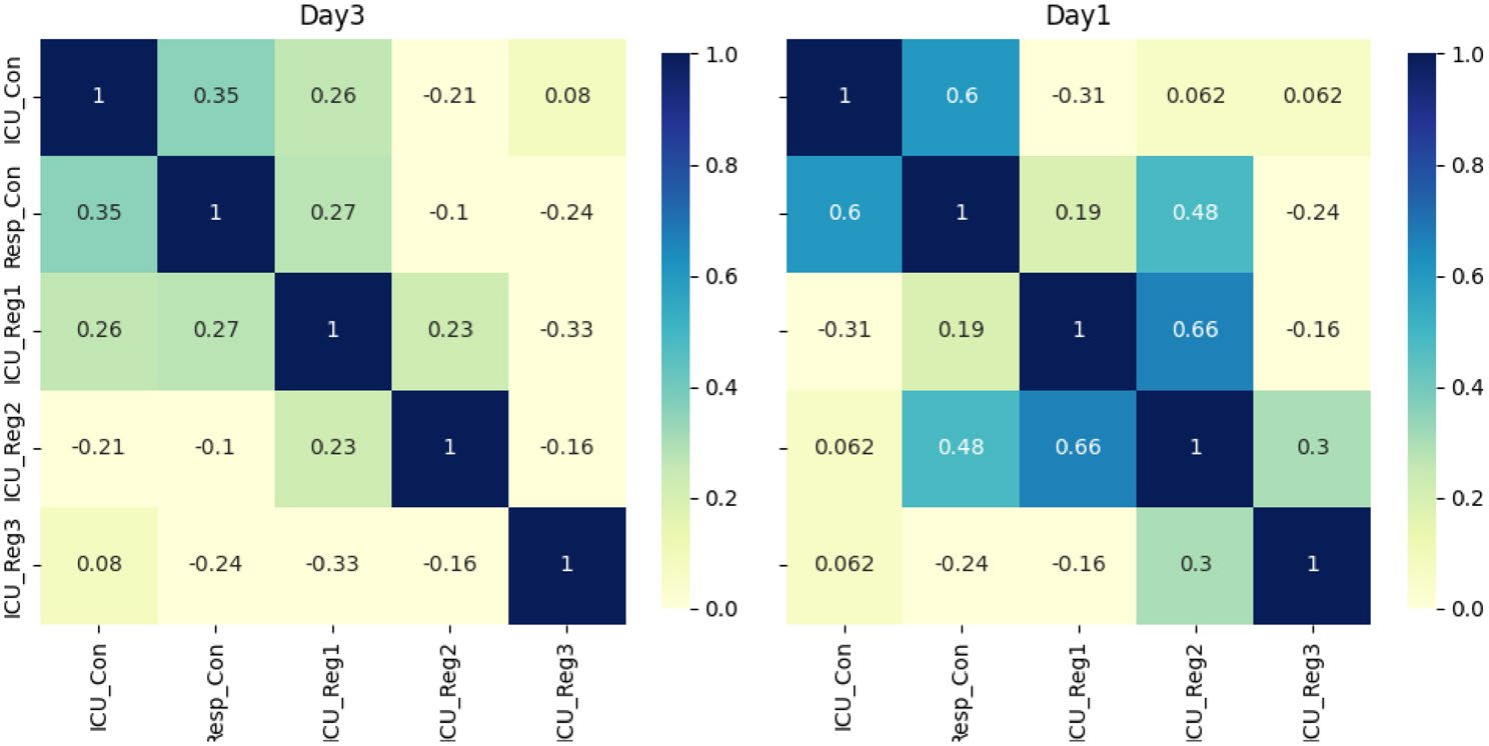
Correlation heatmap between annotators of samples of notes in 3 days, and 1 day prior death

To investigate IAA further, we study correlation of annotator opinions in each of the three groups (admission, discharge, death) and notes not belonging to any groups (notes in the middle). Figure 2, and figures in Supplementary C show the correlation between annotators for notes prior to death, near admission, prior to discharge, and the rest of the notes, respectively.

From the heatmaps, it can be observed that the correlation increases closer to each event (e.g., one day prior to death) and that consistency in opinions in notes prior to death is the highest and most distinguishable with top correlations in (0.48–0.66). The other three groups have less consistency and seem more difficult for annotators, with top correlations in (0.32–0.39).

### Construction of ground truth

Based on the above context, annotators’ labeling cannot be used interchangeably in all samples. Ground truth data should have accumulated evidence supporting validity, similar to the method and logic used by [32].

We consider subsets of data with high Fleiss values as ground truth (5-votes, 4-votes datasets or 5 V, 4 V). 5-votes data has perfect agreement with the Fleiss kappa (k) = 1, the Fleiss kappa for 4-votes dataset is (k) = 0.5451, which reflects moderate agreement according to Fleiss interpretation [23]. Both datasets have p$<$0.0001, rejecting the possibility of occurrence by chance.

While we consider the dataset of 5-votes or 5 V (32 notes) as the perfect candidate for ground truth (gold ground truth), we see that 4-votes or 4 V (110 notes) data as reliable too (silver ground truth). Therefore, the evaluation will be based on these two datasets. Ground truth datasets and other research datasets are described in Table 3.

**Table 3:** Research datasets sizes

Category	Physician	Nursing
Pseudo-labeled training data	10766	10766
Clinicians’ annotated data	156	144
MIMIC Ground Truth (5 V)	17	15
MIMIC Ground Truth (4 V)	46	64
The COVID dataset	23	-

### Evaluation of pseudo-labeling

There are 145 pseudo-labeled notes in the set of 300 annotated notes; 74 notes within 3 days of admission, 48 in the 3 days preceding discharge, and 23 in the 3 days preceding death. However, for the comparison, only notes belonging to groundtruth 5 V and 4 V are extracted. Among those, only notes labeled as improvement or deterioration are extracted from the groups since the pseudo-labels only have two classes. Treating manual annotation as truth, the accuracy of pseudo-labels is computed as a percentage of correct pseudo-labels in the set of groundtruth notes. Percentage where pseudo-labels match with ground truth (5 V,4 V) are calculated using Eq. 2:

2 $$ \text{Percentage of a group }=\frac{\text {CC}}{\text {CBA}}$$

where *CC* is counts of correct pseudo-labels (matched with annotators’ labels) in the group, and *CBA* is counts of all samples labeled as improving or deteriorating by annotators. Using the equation, the percentage of correct labels compared to ground truth datasets (5 V, 4 V) are (100%, 90%) in death group, (100%, 100%) in discharge group, (40%, 42%) in admission group respectively. Table 4 shows counts and percentages of total samples and correctly labeled samples in the three groups.

**Table 4:** Statistics for matching labels between pseudo-labels and gold ground truth (5 V) in middle, and silver ground truth (4 V) on right

			5 V			4 V	
Group	Total Samples	Two-classes	Correct Labels	%	Two-classes	Correct Labels	%
Death	23	2	2	100%	10	9	90%
Discharge	74	9	9	100%	33	33	100%
Admission	48	5	2	40%	12	5	42%

Two-classes are samples labeled as ‘Is deteriorating’, or ‘Is already improving or will get better’ by annotators. ‘%’ is percentage of correct labels compared to ground truth

As shown in Table 4, admission group samples are difficult to judge and validate considering that only 25% of this group can be compared with pseudo-labels. However, this group was kept in the training and evaluation datasets to maintain the diversity of samples and to observe the prediction in different settings. Within certain samples of this group in the ground truth, annotators have reported that a number of patients have exhibited signs of rapid improvement. However, identifying such cases and instances in practice can be challenging and not immediately clear.

Making a final conclusion about the validity of pseudo-labels using the above details is difficult, and further investigations with increased size of annotation samples allowing for statistical significance measures are required. However, the pseudo-labels strongly align with ground truth in samples linked to two major clinical events (discharge, death), presenting validity in these two clusters.

### Model evaluation

Table 5 shows a summary table averaging language models’ accuracies and F1-scores against Kennedy’s lexicon for prediction of clinical sentiment using physician notes (left) and nursing notes (right). Overall, clinical language models outperform Kennedy’s lexicon on ground truth datasets and the COVID data (82% vs 35%) on 5 V, (76% vs 28%) on 4 V, and (80% vs 9%) on the COVID data. With respect to physician notes, all specialized language models achieved high accuracy (82%) for gold ground truth (5 V). Clinical language models have slightly lower accuracy (76%) on the silver ground truth, those with 4 matching opinions (4 V). Predicting sentiment of nursing notes was less accurate (71% on 5 V data and 68% on 4 V) with similar pattern of better accuracy on the gold ground truth (5 V) than on silver ground truth (4 V).

**Table 5:** A comparison of models averaged accuracy (Acc), negative F1-score (N_F1) and positive F1-score (P_F1) across ground truth datasets 5 V, 4 V, and the COVID dataset

Method	Physician notes			Nursing notes		
	5 V	4 V	COVID	5 V	4 V	COVID
Clinical models Acc	0.82	0.76	0.88	0.71	0.68	0.80
Kennedy’s lex Acc	0.35	0.28	0.12	0.40	0.25	0.09
Clinical models N_F1	0.86	0.81	0.83	0.66	0.65	0.68
Kennedy’s lex N_F1	0.63	0.51	0.11	0.57	0.38	0.12
Clinical models P_F1	0.75	0.66	0.89	0.71	0.67	0.75
Kennedy’s lex P_F1	0.22	0.18	0.00	0.50	0.23	0.00

On the COVID dataset [11], clinical language models achieve high accuracy (0.88 on physician notes and 0.80 on nursing notes), whereas Kennedy’s lexicon’s accuracy is poor (0.12, 0.09 respectively).

Supplementary D shows the evaluation for ClinicalT5, SciFive, clinicalBERT, and Kennedy’s lexicon using overall accuracy, and recall, precision, F1-score for positive and negative classes, and applied to assessment and plan sections onward for physicians’ and nursing notes^7^.

During the analysis, it was noticed that accuracy values drop dramatically for samples belonging to patients with short ICU stays (LOS$\le$6), ranging from 24% for Kennedy’s lexicon to 46% for clinical language models. While examining the ground truth samples, it was found that patients with short length of stays (LOS$\le$6) form 60% of the samples within the admission group in the ground truth (3 out of 5 in 5 V admission group, and 7 out of 12 in 4 V admission group), have exhibited signs of rapid improvement, causing inconsistency in the labeling in the training and evaluation. Upon removing these samples from training and evaluation data, the accuracy was increased to 94% and 88% for 5 V and 4 V respectively. Therefore, the model with higher accuracy is released. From the evaluation on ground truth, it can be observed that, except for the group of short stays in ICU, the pseudo-labeling has shown effectiveness in training clinical language models for clinical sentiment classification. This validation of our pseudo-labeling strategy is valuable because it enables the models to be exposed to a large number of training samples in a cost-effective manner, without the cost of manual annotation.

In summary, all language models’ performance align with annotators’ agreement, i.e., higher accuracies are observed for increased agreement on labeling. ClinicalT5 shows higher accuracy scores compared to SciFive and clinicalBERT and all models significantly outperform Kennedy’s lexicon. Models’ performance using physician notes (0.82, 0.76) were higher on 5 V and 4 V compared to nursing notes (0.71, 0.68), respectively. Clinical language models’ performance remains good on the external COVID dataset, suggesting reliable generalizability.

In relation to statistical significance, a normal distribution for accuracy scores was found by Shapiro-Wilk test. Therefore, we use ANOVA test and found statistically significant difference in the means of the accuracy scores (p $<$ 0.05, [95% CI, 0.52, 0.63]). When investigating model performance on all data we found that clinical language models are comparable. Therefore, while ClinicalT5 shows higher accuracy scores in almost all cases compared to SciFive and clinicalBERT, there is no statistically significant difference in the mean accuracies. In contrast, there is a statistically significant difference between all language models and Kennedy’s lexicon (p$<$0.05, [95% CI, −0.47, −0.28]), confirming that their performance differs significantly.

### Zero-shot and few-shot learning by clinical LLMs

The previous experiments rely on training using pseudo-labeling. However, pseudo-labeling is limited to two-classes and uses time-based groups. To expand the exploration of clinical sentiment, we investigate the use of two larger LLMs, pre-trained on neutral or no-sentiment class. We investigate their capability on clinical sentiment, with zero-shot and few-shot settings, on 5 V, 4 V, and COVID datasets. Zero-shot results are presented in Table 6. Models are evaluated directly on the three datasets (including samples with neutral class) with no further training. With respect to few-shot learning, we suggest that samples can be collected after clustering patients into sub-groups using diagnosis or division of ICU such as Medical ICU (MICU) or Neonatal ICU (NICU). This can help models capture the patterns represented in their clinical notes, as ICU patients might have diverse and complicated patterns and are not easily distinguishable by few samples. This was indeed indicated in the results summarized in Table 7. The training samples are six samples collected from 5 V and 4 V datasets. The testing dataset are either sampled from 5 V and 4 V or the COVID dataset. For few-shot using diagnosis, training and testing data are sampled from the three datasets to form a class-balanced training data with the diagnosis of **pneumonia**. Models were trained for three epochs using 5e-5 learning rate.

**Table 6:** Zero-shot accuracy scores of two clinical LLMs using ground truth datasets 5 V, 4 V, and the COVID dataset

Model	Data	Acc
MedGemma	5 V	**0.53**
MedGemma	4V	0.47
MedGemma	COVID	0.24
PMC_LLaMA	5 V	0.10
PMC_LLaMA	4 V	0.18
PMC_LLaMA	COVID	0.32

**Table 7:** Few-shot accuracy scores of two clinical LLMs using samples from ground truth datasets 5 V, 4 V, and the COVID dataset. Mixed samples indicate using all dataset in sampling

Model	Train & Test Data	Acc
MedGemma	Pneumonia (Mixed)	0.43
PMC_LLaMA	Pneumonia (Mixed)	**0.70**
PMC_LLaMA	5 V,4 V	0.33
MedGemma	5 V,4 V	0.50
MedGemma	5 V,4 V & COVID	0.36
PMC_LLaMA	5 V,4 V & COVID	0.14

### Analysis of annotators’ extracts

As mentioned in section “Manual annotation”, part of the manual annotation task was to extract pieces of texts leading to annotators’ selections of labels. We aimed to study how each annotator digests information from the notes and what piece of text helped them make their final decision about a patient’s status or prognosis. We think these extracts have significant value by enabling the following:

- Investigating the most common words or phrases from texts based on a specific patient categorization e.g., patients whom annotators think are doing well.
- Investigating if annotators are building their decisions using the same details.
- Building an ICU-specific sentiment lexicon with support from ICU clinicians.
- Using the extracts to develop AI predictive models and compare the results using the different prediction pipelines.
- Using different prompting e.g., question answering style or few shots.

In this work, we address the first two (i.e., investigating most common words or phrases and studying extracts similarity between annotators), and we keep the rest for future work.

To explore the most repeated words in the extracted text, we use word clouds to visually show the repeated texts in extracts relating to (1) patients whom annotators agree (by majority vote) that their status is improving. (2) patients whom annotators agree (by majority vote) that their status is deteriorating.

**Fig. 3:**
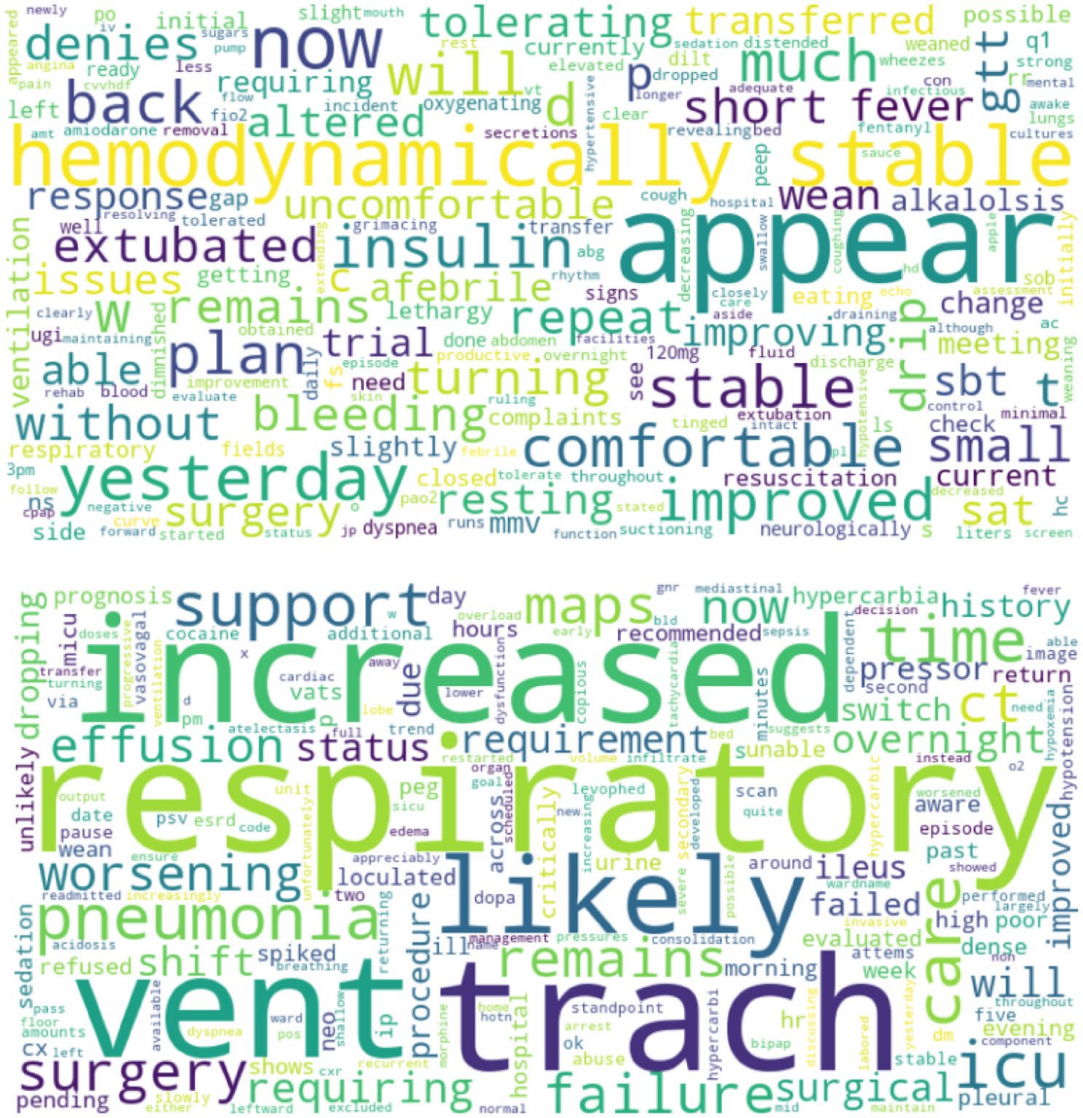
WordClouds for positive notes’ extracts belonging to patients who are believed by majority of physicians to be improving in ICU (above) and negative notes’ extracts belonging to patients who are believed by majority of physicians to be deteriorating in ICU (below)

Figure 3 shows the word clouds for each patient group. In the clouds, the size of each word represents its frequency so the largest word indicates that it is the most frequent. These words can be considered as more indicative of the group more than other less frequent words; e.g., it seems that patients who were deteriorating in the selected samples are likely to have respiratory issues due to the repetition of the words ‘respiratory’, and ‘vent’ (ventilation). On the other hand, ‘extubated’, ‘stable’, and ‘comfortable’ are noticeable on the other group. However, these words may differ depending on the context they were used in. Therefore, we recommend that building any further or subsequent analysis on them e.g., creating a lexicon requires involving a specialized clinician for review and supervision.

To study similarity between extracts, we used three evaluation metrics: ROUGE (Recall-Oriented Understudy for Gisting Evaluation), BLEU (Bilingual Evaluation Understudy), and cosine similarity based on text embeddings. The studies [35, 36] explain both thoroughly. In general, the higher the score and the closer it is to 1, the higher matches of n-grams between the reference and the candidate text. We utilize these metrics to evaluate the similarity between extracts captured by clinicians. We investigated those extracts on the datasets of different votes, and we hypothesize that high votes datasets such as 5 V should have higher scores and lower votes datasets may have lower scores. This hypothesis is based on the fact that annotators might be looking at different aspects while examining notes. Cosine similarity is explained in [37].

**Fig. 4:**
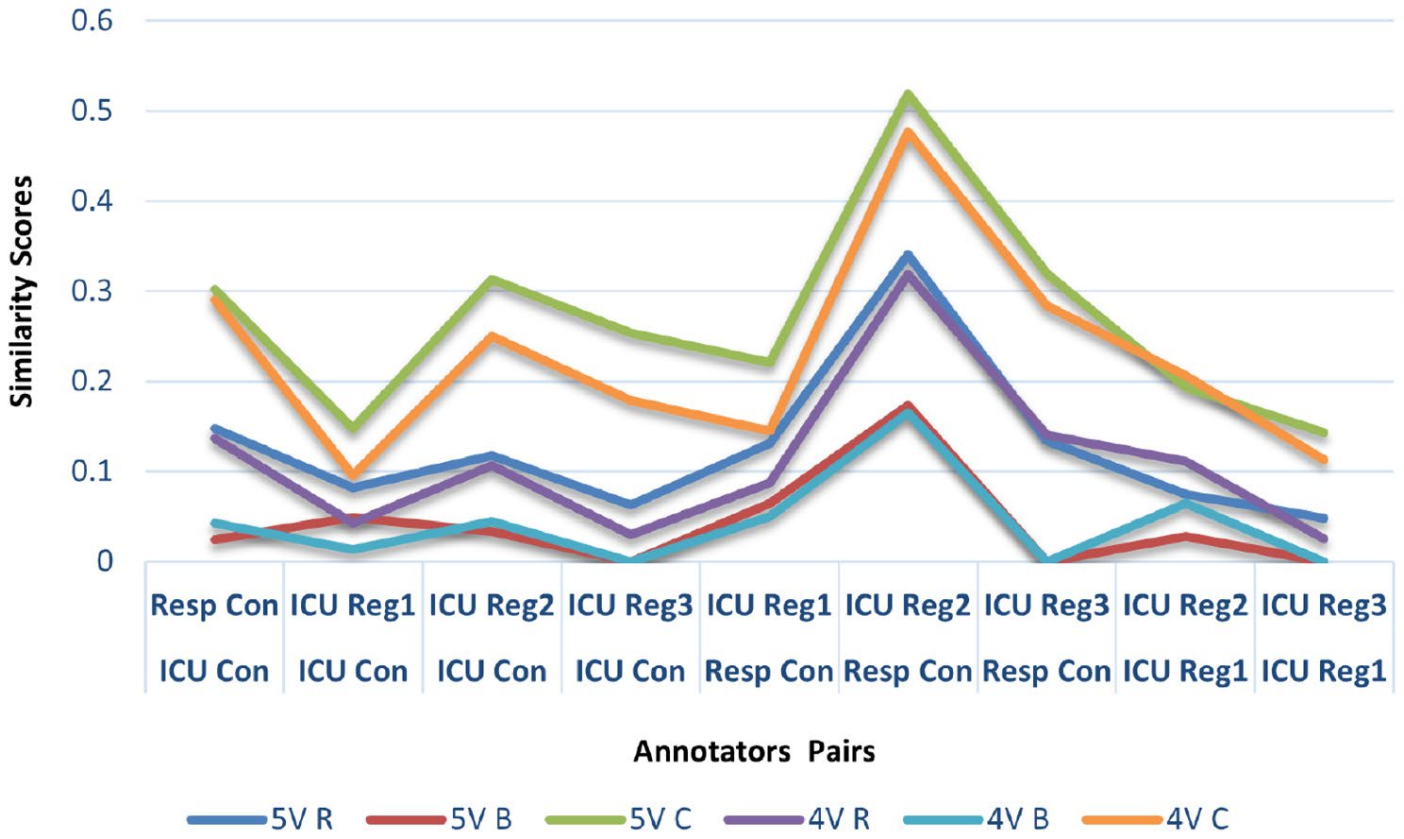
Overall ROUGE-F1, BLEU, cosine similarity scores on groundtruth datasets (5 V, 4 V) comparing texts from annotators’ extracts by pairwise analysis

We used ‘paraphrase-MiniLM-L6-v2’, a Sentence Transformers model [38] for this purpose. Table 8 provides ROUGE (Recall-Oriented Understudy for Gisting Evaluation), BLEU (Bilingual Evaluation Understudy), and cosine similarity mean scores comparing extracts from annotators. Table 8 suggest that our hypothesis is indeed supported by most of the scores (i.e., ROUGE-F1 = 0.12, Bleu = 0.037, Cosine similarity = 0.27 for 5 V dataset vs. 0.11, 0.038, 0.23 for 4 V dataset respectively). Figure 4 shows the scores of extracts’ similarity for all pairs of annotators.

**Table 8:** Average of ROUGE-F1, BLEU, cosine similarity scores on annotators’ extracts

5V_R	5V_B	5V_C	4V_R	4V_B	4V_C
0.122	0.037	0.276	0.111	0.038	0.237

## Challenges and future work

### Manual annotation challenges

This section highlights issues in relation to annotation and ground truth construction and suggests future directions. The annotation task was very challenging. The task requires careful reading of whole notes and comprehending the whole patient’s narrative, while analysing the opinion of notes’ writers without physically examining the patient. Therefore, the task of providing a label for the whole note is not easy compared to fragments of texts as in sentence-based sentiment. Unlike other studies with less informed small text pieces, we think the overall narrative or carefully extracted segments are important to get the complete picture about the patient health status. Fifteen clinicians were recruited over two years but ten quit despite their genuine interest and engagement, due to the difficulty of completing the CITI training to access MIMIC-III (see *PhysioNet*^8^) and annotating the 300 samples. The average time taken per annotator was 8 weeks. The whole manual annotation process took around 15 months. The 300 samples presented to annotators were intended to be a pilot annotation project, but based on the observed difficulty of the work, that set became the final annotation set. This process was very different from a annotation project where tasks are distributed via crowd-sourcing platforms and participation is flexible and open to a wide range of contributors. In contrast, this was a very challenging task with very challenged collaborators with limited time, as evidenced by the fall-off in participants. Therefore, annotation in the clinical field may require an adjustment in annotation tools and additional facilitation and motivation.

As a result of the smaller than intended annotation set, “the three evaluation datasets (4 V, 5 V, and the COVID dataset) are small, with sizes 110, 32, and 23 respectively. (See section “Construction of ground truth”. The COVID data is a secondary dataset used for an initial assessment of the generalizability to other data. Due to unavailability of other public data of clinical notes, the recruitment burden as discussed, and the limited time physicians can provide, only one annotator performed the COVID annotation task, and only 23 samples were annotated. Future work should include expanded datasets and annotation.

With respect to gold ground truth (5 V), and silver ground truth (4 V), the sizes are dependent on inter annotator agreement as described earlier. In such annotation with more than two annotators, the result of inter annotator agreement between all annotators is what influenced the final size for the datasets. This is not something predictable until the whole annotation project is finished, because it is dependent on IAA measures. Hence, in this work, we target 4 or more (5 or full) agreement for high reliability, which reduced the data leading to the small sample sizes. However, for future work, extending by even 1 more annotator could increase the samples in both datasets by reasonable chance because it could add more samples to both by considering the new added agreement. This could be thoughtfully done by collecting subsets of notes with 3 agreement and re-annotate them by the new annotators to see if more agreement can be found for them. Additionally, researchers could rely on Cohen Kappa for pair-wise IAA and construct other versions of ground-truth datasets.

Although the dataset is small now, to our knowledge it is first clinical sentiment dataset and the only one publicly released. In addition, it is quite feasible to do an extension for samples in future, by recruiting extra annotators or by using unsupervised methods like label propagation from the annotated samples to other unlabeled samples in MIMIC-III notes.

The dataset offers the whole notes and assessment plan segment plus small extracts of notes. Therefore, the extracts inherit the labels assigned by clinicians for the whole notes directly because they are what influenced their choice of label. Providing the extracts enables another variant of the clinical sentiment classification, especially for larger LLMs. e.g., classifying sentiment by only using the extracts and comparing the results to whole notes, AssPlan or any other segment of notes.

With the above, the dataset provides rich alternatives for future studies, while doing the heavy lifting (a task of intense and challenging annotation and 15 months of work) in establishing this dataset.

### Handling neutral class

Defining the neutral class in ICUs is challenging, as also noted in previous studies. In fact, within the ICU setting there is no standard definition for any of the three sentiment classes (positive, negative, and neutral), with the neutral category being the most problematic to delineate [2], difficult even for experts. Additionally, the distribution of classes in clinical documents is uneven and biased towards negative or positive classes [8]. The majority class tends to be negative because clinicians continue to mention and document ongoing problems for patients until the patient shows significant improvement. Being focused on documenting noticeable outcomes or changes, clinicians’ notes may not have much of neutral language.

So investigating neutral class requires a clear definition, and further experimentation, enabling training the model for detecting (positive and negative) as one class vs. no-sentiment class. This could be done as a second fine-tuning task using the model and the annotation data and thoughtfully familiarizing the model with the clinical sentiment task using the three classes.

### Additional datasets

The ground truth and the COVID datasets are limited. Larger and additional datasets from different institutions are recommended. MIMIC-III is based on one institution and one country, USA, which may have an impact on generalizing the learning to other settings. The COVID dataset, used for evaluation, is not based on patient records, so assessing the generalizability of this research requires further data. Including additional datasets in the future will further reinforce the conclusion drawn and strengthen its statistical validity.

## Conclusion

In summary, this work introduced the SentimentICUmodel, a model that provides the most effective clinical sentiment for ICU clinical notes. In addition, it presented a guiding comparison for evaluating the effectiveness of several popular models in clinical sentiment classification of ICU notes. Moreover, it introduced the first clinicians’ annotated sentiment dataset for NLP researchers to progress clinical sentiment and other applicable NLP tasks in future work.

## Electronic supplementary material

Below is the link to the electronic supplementary material.

Supplementary Material 1

## Data Availability

Dataset license is available at data host website Physionet^9^. The annotation dataset is currently under-review and will be available for credentialed users of Physionet. Models were trained privately due to restrictions of MIMIC-III data. SentimentICUModel will be available for credentialed users of Physionet with details on implementation and reproducibility.

## Abbreviations

AI: Artificial intelligence
ICU: Intensive care unit
LLMs: Large language models
NLP: Natural Language Processing

## Footnotes

1 Throughout this research, we use these two terms interchangeably and we define clinical sentiment as note writers’ opinion about the patient’s condition and possibly prognosis, whether it is good or bad. See section “Manual annotation” for more details.

2 The dataset is currently under review to be published by Physionet.

3 The model is currently under review to be published by Physionet.

4 The number of annotators is greater than the number of classes to allow for majority votes in case of opinion conflicts.

5 https://www.physionet.org/content/clinical-t5/1.0.0/

6 They have been trained on initial tasks, e.g. masked language modeling (MLM) or next sentence prediction (NSP), which are not related to the tasks here, but we do not know the extent of the effect of the previous training on the results of this research.

7 Note that samples with no assessments or plans are excluded from ground truth 5 V and 4 V in tables.

8 https://physionet.org/content/mimiciii/1.4/
