# Supplementary material for: Clinical sentiment analysis of ICU notes: a dataset and a comparison of lexicon and clinical language model methods
Source: BMC Med Inform Decis Mak. 2026 May 18;26:255. doi: 10.1186/s12911-026-03509-x (PMC13352830; doi:10.1186/s12911-026-03509-x)
Supplement: Supplementary file 1 — Supplementary Material 1 [file 12911_2026_3509_MOESM1_ESM.pdf]

# A Synthesized Note

65-year-old female patient presented to the ED with severe sub-sternal chest pain, known case of CAD with PCI to LCx in 2019. This pain started 30 minutes ago, patient then shocked for suddenly collapsed with VT/VE, then, ECG showed STEMI with significant trop leak. PCI was done and showed 90% lesion in LAD with patent LCx. DES to LCx. Patient recovers well with Trop trending down. TTE: LV with HK, EF is 45-40%. HD is stable during CCU. Ongoing trop trending down.
Assessment: HR: 70 bpm. NSR. Chest discomfort post CPR improving with Tylenol.
Action: Cardiac meds started.
Response: Meds well tolerated.
Plan: Cont. STEMI protocol For discharge today.

# B Cover Note For Annotation Team

## The aim of the research in few sentences

The problem being addressed in this research is the lack of sentiment analysis computational models that can effectively function on ICU clinical texts. The research aims to investigate possible approaches to sentiment analysis that are applicable to the clinical domain to improve their functionality in this domain-specific frame.

## Value

We anticipate that if natural language processing sentiment classification techniques succeeded in recognizing valuable signals from clinical notes, improving prediction in ICU factors will be feasible using these signals. Moreover, clinical sentiment analysis could be utilized in future work to provide a timeline visualization of a patient journey in ICU.

## General definition of sentiment analysis

Identify, extract, or quantify of one or more of the following (Vij and Pruthi 2018):

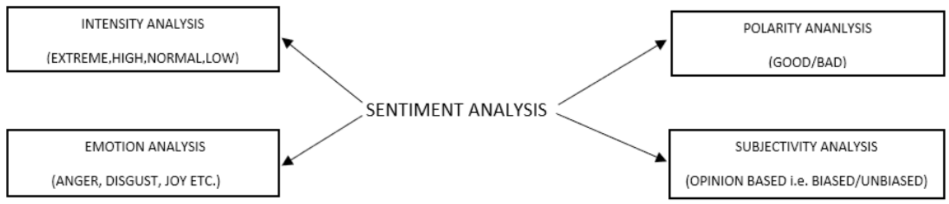


**Fig. 1** Sentiment analysis types (Vij and Pruthi 2018).

This research will focus on opinion and polarity analysis as explained next.

## Definition of Sentiment analysis in ICU notes

We define clinical sentiment as notes writers' opinion about the patient's condition and possibly prognosis, whether it's good, bad, neutral, or not mentioned. In other words, the question we aim to answer is:

Do you think that the person who wrote this note believes the patient's health:

- Is already improving or will get better?
- Is deteriorating?
- Is neither improving nor deteriorating?
- OR that there is no indication of the note writer's opinion.

## Dataset

The dataset used in this study is the MIMIC-III database, which contains de-identified information about patients of the critical care units at Beth Israel Deaconess Medical Center in Boston, Massachusetts, USA in the period 2001-2012 (Johnson et al. 2016), (Johnson, Alistair, Pollard, Tom, and Mark, Roger 2015), (Goldberger et al. 2000).

## Motivation for manual labeling

Manually labeled data is an important component for evaluating our sentiment models. Specifically, we need a set of notes whose "sentiment" has been assigned manually by expert annotators to test our sentiment models. No such labelled datasets of ICU notes exist. Because clinical language is so specialized, this manual labelling can only be done by clinical experts like you. We are initially aiming for 300 labelled notes.

## Task Description

We are asking you to read a note and choose a label in the list of column D, namely "Overall label", that reflects the patient's status. e.g., **The clinician believes that this patient is already improving or will get better**, when a note indicates that the patient is ok, has made some progress or expected to get better, **The clinician believes that this patient is deteriorating**, when a note indicates that the patient's condition has deteriorated, **The clinician believes that this patient is neither improving nor deteriorating** when a note indicates no specific direction to improvement or deterioration, and **There is no indication of the note writer's opinion** when no opinion can be recognized from the note**.**

If possible, and where there are clear indicators in the text that lead to your decision of label, we would appreciate if you could copy those words or sentences into column E, namely "Extracts supporting the selection of label". These extracts might allow us to enhance our sentiment models.

## Instructions for annotation team

1. The annotation file is encrypted to keep it secure for transmission and storage. The password will be shared with you prior to the task.
2. The annotation task should be completed by each physician independently.

3. The annotation is meant to target and capture the impression that is expressed by the clinicians who wrote the note (i.e., their opinion) about the patient. It is not meant to test or evaluate the clinical decision capabilities of the annotators.
4. Data was extracted automatically by a programming script. If an annotator finds anything that appears to be an error, please let me know before completing the task.
5. There are some repetitive headers in the notes potentially due to copy and paste by note writers.
6. When you complete the task, please add your first name to the file name i.e., file name should look like: Annotation file – your first name.

## The required field, and a desirable extract field

1. **Overall label** is the main field in this annotation task. This should reflect the status of the patient as expressed in the note.
2. **Extracts supporting the selection of label** . These should be copies from notes text (i.e., a sentence, or a phrase of a set of words but not a paragraph) reflecting a specific condition associated with improvement, deterioration, no-change or none. Please separate multiple extracts and start each new extract with double dashes (–).

The following describe possible examples of this field:

1. Extracts associated with **improvement.** This should contain copied phrases that reflect any improvement in a patient's condition, e.g., "extubated [**4-13**], and improving on antibiotic therapy.", "normal blood pressure, not on pressors."
2. Extracts associated with **no change in status**. This should contain copied phrases that reflect stability in specific aspects of a patient's condition, e.g. "Remains intubated mechanically ventilated."
3. Extracts associated with **deterioration**. This should contain copied phrases that reflect any deteriorating aspects of a patient's condition, e.g., "no viable therapeutic options that seem feasible for living outside of intensive care"

## Deidentification by owners of MIMIC-III data

MIMIC-III data was changed by owners in compliance with HIPPA.

1. Patients' names, doctors' names, telephone numbers, addresses, dates are all replaced by tokens surrounded by [** and **], e.g., [**Last Name**].
2. "Dates were shifted into the future by a random offset for each individual patient in a consistent manner to preserve intervals, resulting in stays which occur sometime between the years 2100 and 2200." [1]
3. Ages are also modified. "Dates of birth for patients aged over 89 were shifted to obscure their true age and comply with HIPAA regulations: these patients appear in the database with ages of over 300 years."[2]

# References

Goldberger, A. L., L. A. Amaral, L. Glass, J. M. Hausdorff, P. C. Ivanov, R. G. Mark, J. E. Mietus, G. B. Moody, C. K. Peng, and H. E. Stanley. 2000. "PhysioBank, PhysioToolkit, and PhysioNet: Components of a New Research Resource for Complex Physiologic Signals." *Circulation* 101 (23): E215-220. https://doi.org/10.1161/01.cir.101.23.e215.

Johnson, Alistair E. W., Tom J. Pollard, Lu Shen, Li-wei H. Lehman, Mengling Feng, Mohammad Ghassemi, Benjamin Moody, Peter Szolovits, Leo Anthony Celi, and Roger G. Mark. 2016. "MIMIC-III, a Freely Accessible Critical Care Database." *Scientific Data* 3 (1): 160035. https://doi.org/10.1038/sdata.2016.35.

Johnson, Alistair, Pollard, Tom, and Mark, Roger. 2015. "MIMIC-III Clinical Database." PhysioNet. https://doi.org/10.13026/C2XW26.

Vij, Anneketh, and Jyotika Pruthi. 2018. "An Automated Psychometric Analyzer Based on Sentiment Analysis and Emotion Recognition for Healthcare." *Procedia Computer Science*, International Conference on Computational Intelligence and Data Science, 132 (January): 1184–91. https://doi.org/10.1016/j.procs.2018.05.033.

[1] https://physionet.org/content/mimiciii/1.4/

[2] https://physionet.org/content/mimiciii/1.4/

# C Additional Correlation Heatmaps for Annotation Process

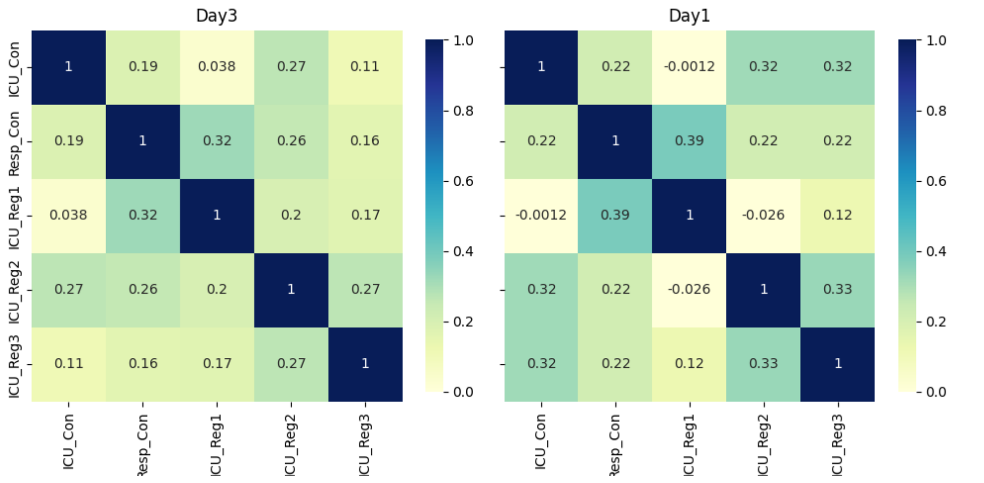


**Fig. 2** Correlation heatmap between annotators of samples of notes in 3 days, and 1 day after admission.

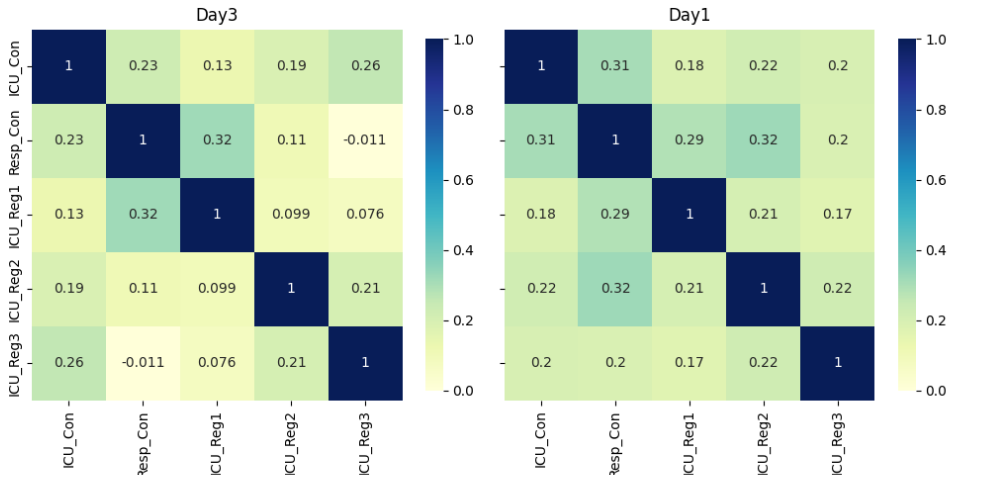


**Fig. 3** Correlation heatmap between annotators of samples of notes in 3 days, and 1 day prior discharge.

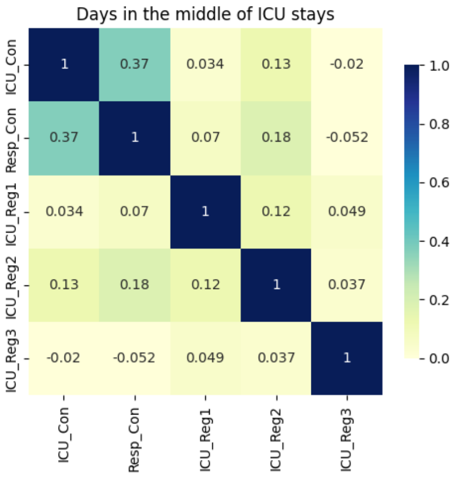


**Fig. 4** Correlation heatmap between annotators of samples of notes that don't belong to admission, death, or discharge groups.

# D Additional Tables

**Table 1** Counts matrix for annotators' labeling on the manual annotation data. Negative label is "deteriorating" while positive label is "already improving or will get better". Neither is "neither improving nor deteriorating" and No_opinion is "that there is no indication of the note writer's opinion".

| **Annotator** | **negative** | **positive** | **neither** | **no_opinion** |
|---|---|---|---|---|
| ICU-C | 98 | 75 | 109 | 18 |
| Resp-C | 80 | 135 | 38 | 47 |
| ICU-R1 | 101 | 117 | 48 | 34 |
| ICU-R2 | 130 | 154 | 12 | 4 |
| ICU-R3 | 119 | 75 | 76 | 30 |

**Table 2** Evaluation on physicians' notes of ClinicalT5, SciFive, clinicalBERT, and Kennedy's-lexicon using overall accuracy, and recall, precision, F1-score for positive and negative classes, and applied to assessment and plans sections onwards.

| Model | Category | Size | Acc | Neg_R | Neg_P | Neg_F1 | Pos_R | Pos_P | Pos_F1 |
|---|---|---|---|---|---|---|---|---|---|
| ClinicalT5 | 5V | 17 | 0.8235 | 0.8000 | 0.8889 | 0.8421 | 0.8571 | 0.7500 | 0.8000 |
| ClinicalT5 | 4V | 45 | 0.8000 | 0.8462 | 0.8148 | 0.8302 | 0.7368 | 0.7778 | 0.7568 |
| ClinicalT5 | Covid | 23 | 0.8261 | 0.8571 | 0.6667 | 0.7500 | 0.8125 | 0.9286 | 0.8667 |
| Scifive | 5V | 17 | 0.8235 | 1.0000 | 0.7692 | 0.8696 | 0.5714 | 1.0000 | 0.7273 |
| Scifive | 4V | 45 | 0.7333 | 0.9231 | 0.7059 | 0.8000 | 0.4737 | 0.8182 | 0.6000 |
| Scifive | Covid | 23 | 0.8696 | 0.7143 | 0.8333 | 0.7692 | 0.9375 | 0.8824 | 0.9091 |
| ClinicalBERT | 5V | 17 | 0.8235 | 1.0000 | 0.7692 | 0.8696 | 0.5714 | 1.0000 | 0.7273 |
| ClinicalBERT | 4V | 45 | 0.7556 | 0.9231 | 0.7273 | 0.8136 | 0.5263 | 0.8333 | 0.6452 |
| ClinicalBERT | Covid | 23 | 0.9565 | 1.0000 | 0.9412 | 0.9697 | 0.8571 | 1.0000 | 0.9231 |
| Kennedy's lex | 5V | 17 | 0.3529 | 0.5000 | 0.8333 | 0.6250 | 0.1429 | 0.5000 | 0.2222 |
| Kennedy's lex | 4V | 45 | 0.2826 | 0.4231 | 0.6471 | 0.5116 | 0.1053 | 0.6667 | 0.1818 |
| Kennedy's lex | Covid | 23 | 0.1200 | 0.0625 | 1.0000 | 0.1176 | 0.0000 | 0.0000 | 0.0000 |

**Table 3** Evaluation on nursing notes of ClinicalT5, SciFive, clinicalBERT, and Kennedy's lexicon using overall accuracy, and recall, precision, F1-score for positive and negative classes, and applied to assessment and plans sections onwards.

| Model | Category | Size | Acc | Neg_R | Neg_P | Neg_F1 | Pos_R | Pos_P | Pos_F1 |
|---|---|---|---|---|---|---|---|---|---|
| ClinicalT5 | 5V | 14 | 0.7143 | 0.6000 | 0.6000 | 0.6000 | 0.7778 | 0.7778 | 0.7778 |
| ClinicalT5 | 4V | 58 | 0.7241 | 0.6818 | 0.6250 | 0.6522 | 0.7500 | 0.7941 | 0.7714 |
| ClinicalT5 | Covid | 23 | 0.8261 | 0.8571 | 0.6667 | 0.7500 | 0.8125 | 0.9286 | 0.8667 |
| Scifive | 5V | 14 | 0.7857 | 0.8889 | 0.8000 | 0.8421 | 0.6000 | 0.7500 | 0.6667 |
| Scifive | 4V | 58 | 0.6897 | 0.7222 | 0.7647 | 0.7429 | 0.6364 | 0.5833 | 0.6087 |
| Scifive | Covid | 23 | 0.7826 | 0.2857 | 1.0000 | 0.4444 | 1.0000 | 0.7619 | 0.8649 |
| ClinicalBERT | 5V | 14 | 0.6429 | 0.6000 | 0.5000 | 0.5455 | 0.6667 | 0.7500 | 0.7059 |
| ClinicalBERT | 4V | 58 | 0.6207 | 0.6818 | 0.5000 | 0.5769 | 0.5833 | 0.7500 | 0.6562 |
| ClinicalBERT | Covid | 23 | 0.7826 | 0.9375 | 0.7895 | 0.8571 | 0.4286 | 0.7500 | 0.5455 |
| Kennedy's lex | 5V | 14 | 0.3571 | 0.4000 | 1.0000 | 0.5714 | 0.3333 | 1.0000 | 0.5000 |
| Kennedy's lex | 4V | 58 | 0.1897 | 0.2727 | 0.6000 | 0.3750 | 0.1389 | 0.6250 | 0.2273 |
| Kennedy's lex | Covid | 23 | 0.0435 | 0.0625 | 1.0000 | 0.1176 | 0.0000 | 0.0000 | 0.0000 |
